# Thapsigargin Induces Apoptosis by Impairing Cytoskeleton Dynamics in Human Lung Adenocarcinoma Cells

**DOI:** 10.1155/2014/619050

**Published:** 2014-01-28

**Authors:** Fei Wang, Da-zhong Liu, Hao Xu, Yi Li, Wei Wang, Bai-lu Liu, Lin-you Zhang

**Affiliations:** ^1^Department of Thoracic Surgery, The Second Affiliated Hospital of Harbin Medical University, No. 246 Xuefu, Nangang District, Harbin 150086, China; ^2^Department of Computerized Tomography, The Second Affiliated Hospital of Harbin Medical University, No. 246 Xuefu, Nangang District, Harbin 150086, China

## Abstract

The objective of this study was performed to investigate the effects of thapsigargin on apoptosis, actin cytoskeletal dynamics, and actin cytoskeletal proteins in human lung adenocarcinoma cell. Thapsigargin is a specific irreversible inhibitor of ER calcium-ATPase, which may promote ER stress by depletion of lumenal calcium stores and show potential to induce cell death. The effects of thapsigargin on the apoptosis in A549 cells were assayed by Hoechst staining. Moreover, the F-actin staining by Rhodamine-phalloidin and RhoA antibody for cytoskeleton organizations were applied to A549 cells. To confirm the impairment of cytoskeletal dynamics treated with thapsigargin, western blots were applied to analyze the protein levels of p-Cofilin-1 (Ser3), Cofilin-1, and pPaxillin (Tyr118), as well as RhoA and pS6 (S240/244). Results suggest that thapsigargin may induce cell death in A549 cells with a time- and dose-dependent manner. The F-actin fibers and RhoA signals are also reduced with a time- and dose-dependent manner by thapsigargin treatment. The phosphorylation forms of Cofilin-1 and paxillin are attenuated by 1 **μ**M thapsigargin treatment for 24 h. These alternations may be caused by the inhibition of of mTORC1 activities (indicated by pS6 (Ser240/244)) and RhoA pathways after thapsigargin treatment. The present findings highlight important roles of calcium entry in cytoskeleton organization and apoptosis in human lung adenocarcinoma cells and will help to set a stage to the clinical treatment of cancer cell metastasis.

## 1. Introduction

Cytoskeleton is required for many biological processes, such as embryonic morphogenesis, immune surveillance, tissue repair, and regeneration. Aberrant regulation of cytoskeleton dynamics drives progression of cancer invasion and metastasis [1, 2]. Cancer cell metastasis is a multistage process involving invasion into surrounding tissue, intravasation, transit in the blood or lymph, extravasation, and growth at a new site. Many of these steps require cell motility, which is driven by cycles of actin polymerization and depolymerization [3]. Actin networks consist of branched or linear filaments and regulate many essential cellular processes. The actin cytoskeleton functions in the generation and maintenance of cell morphology and polarity and in endocytosis, intracellular trafficking, contractility, motility, and cell division. The assembly and disassembly of actin filaments, as well as their organizations into functional higher-order networks, are regulated by several actin-binding proteins, many of which are conserved from yeast to human [4, 5].

Malignant cancer cells utilize their intrinsic migratory ability to invade adjacent tissues and the vasculature and ultimately to metastasize. The motility of tumor cells is driven by the polymerization of actin monomers into polarized filaments. These filaments, termed F-actin, are in a constant state of flux with new monomers being added [6, 7]. One of the proteins identified in F-actin formation is Cofilin-1. Cofilin-1 is an actin-depolymerizing factor and regulates actin cytoskeletal reorganization. Phosphorylation of Cofilin-1 on Serine-3 is known to block these activities, which is regulated by RhoA GTPases through mTORC1-RhoA-Limk-Cofilin-1 pathways [8, 9]. Earlier results have been coupled with intracellular calcium influx and cytoskeletal dynamic *in vitro* and* in vivo*. For example, calcium influx into rat brain synaptosomes causes the breakdown of F-actin under the plasma membrane [10–12]. And inhibition of actin polymerization has a biphasic time-dependent effect on calcium entry, showing an initial potentiation followed by inhibition of calcium entry [13]. Moreover, thapsigargin, a specific irreversible inhibitor of ER calcium-ATPase, has been used to investigate the effect of a disturbed endoplasmic reticulum (ER) calcium homeostasis on different processes of cells, including cytoskeleton dynamics. It is interesting that thapsigargin-induced depletion of ER calcium stores severely inhibits F-actin contents in human monocytic cells [14]. Thus, thapsigargin may prove to be a useful tool for investigating calcium influx and cytoskeleton dynamics underlying cell apoptosis.

Although progress has been made, it is still largely unknown whether the calcium influx couples with cytoskeletal dynamics and affects cell apoptosis, especially in cancer cells. To examine the critical role between calcium signaling, cytoskeleton, and cell apoptosis, we focus on the effect of ectopic entry of calcium influx by thapsigargin on actin cytoskeleton apoptosis in A549 human lung adenocarcinoma cell lines. Our findings demonstrate that thapsigargin treatment may induce cell death in A549 cells in a time- and dose-dependent manner. For ectopic entry of calcium influx being induced thapsigargin, we find that the cytoskeletal dynamics is impaired by thapsigargin treatment, indicated by F-actin staining and biochemical evidence. The effects of thapsigargin on cell death in A549 tumor cells may be mediated by mTORC1-RhoA-Cofilin-1 pathway, because thapsigargin treatment may dramatically inhibit mTORC1 activity and RhoA protein level. Our work will help to understand a novel clue regarding the relationship between calcium signaling, cytoskeleton, and cell apoptosis in A549 tumor cells.

## 2. Materials and Methods

### 2.1. Chemicals and Materials

The inhibitor of ER calcium-ATPase thapsigargin was purchased from Sigma-Aldrich (St. Louis, MO, USA). Dulbecco's Modified Essential Medium (DMEM), Fetal Bovine Serum (FBS), and F-actin probes of Rhodamine Phalloidin R415 were purchased from Gibco Invitrogen Corporation (Carlsbad, CA, USA). The Hoechst kit was from Beyotime Biotechnology Co. (Haimen, Jiangsu, China). The following antibodies, anti-p-Cofilin-1, anti-Cofilin-1, and anti-RhoA were from Santa Cruz Biotechnology (Santa Cruz, CA, USA). The p-Paxillin (Tyr118) and pS6 (Ser240/244) antibodies were from Cell Signaling Technology (Danvers, MA, USA) and anti-GAPDH was from Millipore (Billerica, MA, USA). Other chemicals were of the highest purity available.

### 2.2. Assays of Cell Culture

A widely used human lung adenocarcinoma cell line A549 was chosen as *in vitro* experiment system, which was obtained from Shanghai Institute of Cell Biology (Introduced from American Type Culture Collection). A549 cells were derived from a lung adenocarcinoma and widely used to study the amplification process in tumors. In the present study, A549 cells were plated in 6-well plates at 1.0 × 10^6^ cells/mL. The cells were incubated in Dulbecco's Modified Essential Medium (DMEM) containing 10% Fetal Bovine Serum (FBS) plus antibiotics for 24 h in 5% CO_2_ at 37°C.

### 2.3. Pharmacological Manipulations

For ectopic calcium influx in A549 cells, the final concentrations (1, 100, and 1000 nM) of thapsigargin were applied to these cells and then incubated from 6 h to 24 h. No additives were used as internal controls. After culturing, the cells were harvested for subsequent Hoechst stainings and immunostainings. To further study the role of thapsigargin on cytoskeleton molecules in A549 cells, thapsigargin of 1 *μ*M for 6 h was applied to A549 cells for biochemical examinations.

### 2.4. Assay of Hoechst Staining

For the preparation of Hoechst staining, A549 cells were plated with 1.0 × 10^5^ cells/mL in 6-well plates. After pharmacological manipulations, cells were directly stained with Hoechst kit from Beyotime. The cell counting was carried out through the use of National Institutes of Health software ImageJ, which is available at http://rsbweb.nih.gov.

### 2.5. Assay of F-Actin Staining and Immunostaining

To detect the F-actin fibers in thapsigargin-treated A549 cells, the Rhodamine Phalloidin R415 probes were applied to stain the F-actin fibers. For the preparation of Rhodamine Phalloidin staining, A549 cells were plated with 1.0 × 10^5^ cells/mL in 6-well plates. After thapsigargin-treated at different concentrations from 6 h to 24 h, cells were fixed with 4% Paraformaldehyde (PFA) and 4% sucrose in phosphate-buffered saline for 30 min and then permeabilized with 0.25% Triton-X 100 in PBS for another 5 min at room temperature. Anti-RhoA antibody was used to detect RhoA signals in these cells. After washing extensively, Rhodamine Phalloidin R415 probes were added into these cells, as well as antibodies-Alexa Fluor 594 goat anti-mouse IgG (Invitrogen, Carlsbad, CA). Then after a second washing for three times, the cover slips were mounted onto glass slides with antifade reagent with 4′,6-diamidino-2-phenylindole (DAPI) for nuclear labellings.

### 2.6. Assay of Western Blots

To extract proteins, cultured A549 cells were sonicated with lysis buffer (PBS with 1% Triton X-100 and protease inhibitors). The cell lysate supernatants were harvested by centrifugation at l0000 rpm for 10 min at 4°C. Protein concentrations of the cell supernatants were evaluated and measured by BCA Protein Assay kit (Thermo Fisher Scientific Inc., Rockford, IL, USA). Equal amounts of the proteins from each extract were separated in a SDS-polyacrylamide gel (12.6%) with 5% stacking gel in SDS-Tris-glycine running buffer. The proteins were then transferred electrophoretically using a PVDF membrane by standard procedures. The membranes were then blocked by 5% nonfat dry milk in PBST (PBS with 0.1% Tween 20, pH 7.6) for 1 h at room temperature and probed overnight by proper primary antibodies diluted in PBST at 4°C. The membranes were rinsed 3 times with PBST and incubated with proper secondary antibodies diluted in PBST for 1 h at room temperature. Then, the membranes were rinsed another 3 times with PBST at room temperature for 10 min, and proteins were detected by Super Signal enhanced chemiluminescence development (ECL) (Thermo Scientific Pierce) reagent and exposed to films (Kodak). The protein level quantification was carried out by ImageJ.

### 2.7. Statistical Analysis

All statistical analysis was performed by Image software. Quantitative data were shown in *x* ^−^ ± *s* using *t*-tests for comparisons. The values 0.05 (*), 0.01 (**), and 0.001 (***) were assumed as the level of significance for the statistic tests carried out.

## 3. Results

### 3.1. Thapsigargin Induces Cell Apoptosis in A549 Cells

**Figure 1:**
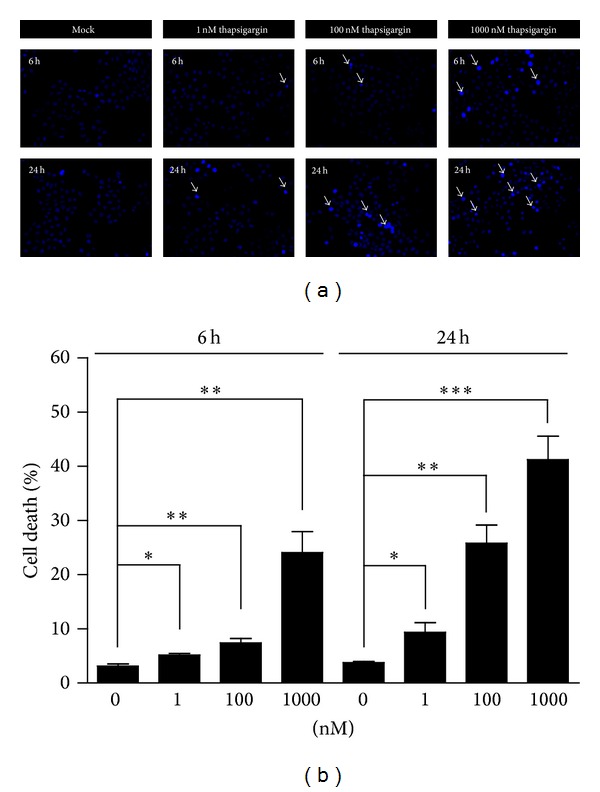
Thapsigargin induces cell apoptosis in A549 cells. (a) Hoechst staining (blue) showing the increasing of apoptotic A549 cells by thapsigargin (1 nM, 100 nM, and 1 *μ*M) treatment for 6 and 24 h (white arrows). (b) Histograms showing the quantification of the cell death (%) in A549 cells after thapsigargin treatment. Results are averages of three independent experiments. Data represent mean ± SEM. *P < 0.05, **P < 0.01, and ***P < 0.001.

To examine whether the thapsigargin treatment may induce the cell death in A549 cells, we applied Hoechst staining to A549 cells treated by thapsigargin (Figure 1). The results show that thapsigargin has slight effects on cell death at the final concentration of 1 nM (cell death by 5.2%) or 100 nM (by 7.4%) for 6 h treatment (Figure 1(b)). The percentage of cell death increases significantly to 24.1% at the concentration of 1 *μ*M (Figure 1(b)). To examine whether the effects of thapsigargin on cell death of A549 cells is time-dependent or not, we prolonged the treated time of thapsigargin on A549 cells to 24 h. Our results suggest that the percentages of cell death increase to 9.4% (1 nM), 25.8% (100 nM), and 41.2% (1 *μ*M) after thapsigargin treatment (Figure 1(b)). These findings support the notion that thapsigargin may induce cell death in A549 cells in a time- and dose-dependent manner.

### 3.2. Thapsigargin Impairs Actin Cytoskeleton Organizations in A549 Cells

**Figure 2:**
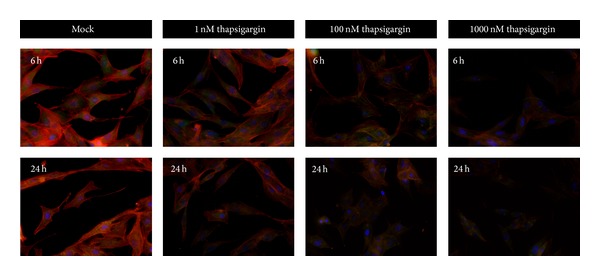
Thapsigargin impairs actin cytoskeleton organizations in A549 cells. The reductions of F-actin fibers by thapsigargin (1 nM, 100 nM, and 1 *μ*M for 6 and 24 h) treatments were shown by immunostaining. Red fluorescence is indicated by Rhodamine-phalloidin probes, green by RhoA antibody, and blue by DAPI.

To study the cellular mechanisms of how thapsigargin induces cell death in A549 cells, we focused on the cytoskeletal dynamics, because we noted that A549 cells tended to shrink after thapsigargin treatment (Data not shown). Thus, we carried out F-actin staining by Rhodamine labeled Phalloidin probes in A549 cells. Being consistent with Hoechst stainings, our results show that the F-actin fibers are reduced in a time- and dose-dependent manner after thapsigargin treatment (Figure 2). Moreover, RhoA signals, indicated by the greed-fluorescence, are also reduced after thapsigargin treatment, in parallel with Rhodamine-phalloidin signals, while DAPI signals labelled blue indicate the nuclear locations (Figure 2). The parallel reduction of F-actin and RhoA signals by thapsigargin treatment confirms that thapsigargin may impair the cytoskeleton dynamics and organizations.

### 3.3. Thapsigargin Disrupts Actin Cytoskeletal Proteins in A549 Cells

**Figure 3:**
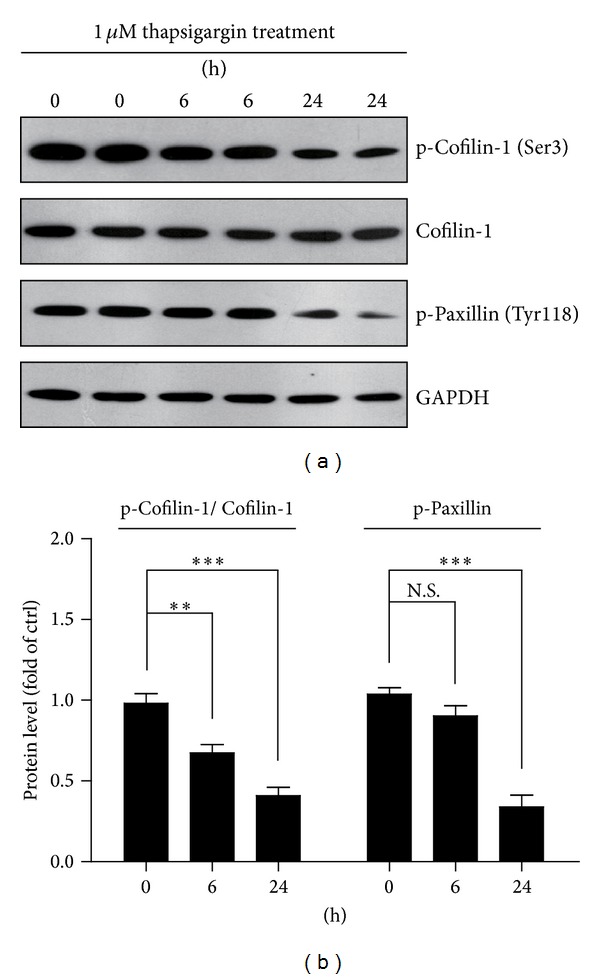
Thapsigargin disrupts actin cytoskeletal proteins in A549 cells. The reductions of protein levels of p-Cofilin-1 (Ser3) and p-Paxillin (Tyr118) by thapsigargin treatment (1 *μ*M for 6 and 24 h) in A549 cells were shown by Western blots (a) and histograms (b). Notes showed that the protein level of total Cofilin-1 is not affected by thapsigargin treatment. Results are averages of four independent experiments. Data represent mean ± SEM. **P < 0.01, ***P < 0.001, N.S, and no statistical difference.

To confirm the impairment of cytoskeletal dynamics in thapsigargin treated A549 cells, we examined the cellular pathways regulating F-actin organizations. By western blots, we show that Cofilin-1 phosphorylations are reduced after thapsigargin treatment, while the total protein levels of Cofilin-1 are not altered (Figure 3(a)). The ratio of p-Cofilin-1 to Cofilin-1 reduces to 67.4% after thapsigargin treatment of 1 *μ*M for 6 h, and to 40.9% after 1 *μ*M for 24 h (Figure 3(b)). Consistently, the phosphorylation of paxillin is also reduced to 33.9% after 1 *μ*M thapsigargin treatment for 24 h (Figures 3(a) and 3(b)). It is of note that the protein level of p-Paxillin is not altered with statistical difference after 1 *μ*M thapsigargin treatment for 6 h (Figures 3(a) and 3(b)). This may be due to the delay effect of thapsigargin on regulator molecules of cytoskeletons. Taken together, our findings suggest that thapsigargin treatment may impair the balance of cytoskeletal dynamics, depolymerizing actin fibers, and inhibiting actin reorganizations.

### 3.4. Thapsigargin Impairs Cytoskeletal Dynamics via mTOR-RhoA Pathways in A549 Cells

**Figure 4:**
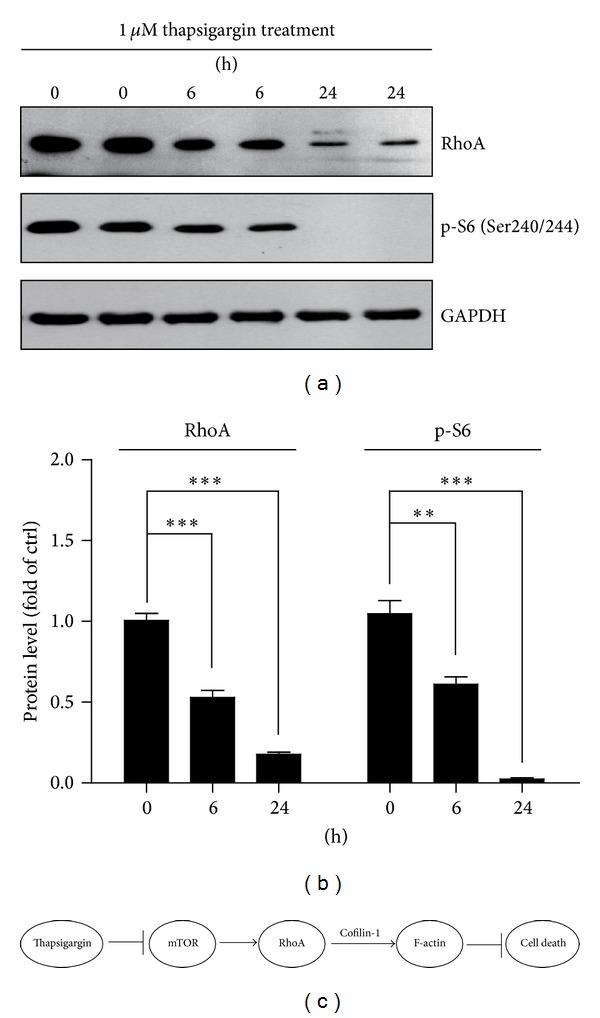
Thapsigargin impairs cytoskeletal dynamics *via* mTOR-RhoA pathways in A549 cells. The changes in the protein levels of RhoA and pS6 (S240/244) by thapsigargin treatment (1 *μ*M for 6 and 24 h) in A549 cells were shown by Western blots (a) and histograms (b). Results are averages of four independent experiments. Data represent mean ± SEM. **P < 0.01, ***P < 0.001. (c) Schematic representation highlighting the models of thapsigargin inducing cell apoptosis by impairing actin cytoskeletons in A549 cells. Thapsigargin may induce cell death in A549 cells, by disrupting the actin cytoskeleton organizations, which is mediated by inhibiting mTOR-RhoA-Cofilin-1 pathways.

To study the molecular mechanism of thapsigargin's effect on cytoskeleton dynamics, the protein levels of mTORC1 indicators and downstream factor RhoA were examined. Our results reveal that the protein levels of pS6 (Ser240/244), a well-known indicator of mTORC1 activity, are reduced to 61.1% (1 *μ*M for 6 h) and non-detectable (1 *μ*M for 24 h) after thapsigargin treatment (Figures 4(a) and 4(b)). Subsequently, the protein levels of RhoA, a key regulator of actin cytoskeletal dynamics, are also reduced to 52.9% (1 *μ*M for 6 h) and 17.7% (1 *μ*M for 24 h) after thapsigargin treatment (Figures 4(a) and 4(b)). The reduction of RhoA protein levels is consistent with previous reduction of signals of RhoA immunostaining after thapsigargin treatment (Figure 2). These results indicated that thapsigargin may impair cytoskeletal dynamics through mTOR-RhoA pathways in A549 cells.

## 4. Discussion

Cancer progression is a multistep process that enables tumor cells to disperse to points far from a given primary tumor mass, and this often leads to metastasis. Cell movement through tissue thus plays a crucial and primary role in cancer progression. This process requires a series of distinct but concerted biological events in which the actin cytoskeleton plays essential roles [5, 15]. In decades, our understanding of the molecules involved in regulating actin cytoskeletal dynamics has increased. Thapsigargin has been reported to induce cell death in several tumor cell lines, by either increasing the store-mediated calcium entry or ER stress [16–18]. It has been reported that thapsigargin treatment rapidly induce a sustained increase in calcium concentration and DNA fragmentation and induce cell death by altering cell morphology or activating apoptotic pathways [14, 19]. In the present study, we demonstrate that thapsigargin, a specific irreversible inhibitor of ER calcium-ATPase, induces cell death by impairing the cytoskeletal dynamics and actin organizations in A549 human lung adenocarcinoma cell line. This process may be mediated by the mTOR-RhoA-Cofilin-1 pathways, because thapsigargin treatment may dramatically inhibit mTORC1 activity, and reduce RhoA proteins and attenuating Cofilin-1 phosphorylations (Figure 4(c)). mTOR is a central controller of cell proliferation, growth, and survival and functions in cells at least as two complexes, mTORC1 and mTORC2. It has been reported that mTOR pathways regulate tumor cell migration and cancer metastasis. For example, rapamycin suppresses IGF-1 stimulated F-actin reorganization and migration in various tumor cell lines by inhibiting mTORC1 activity. Rapamycin may also inhibit F-actin reorganization and cell motility by downregulation of RhoA protein expression and activity [20, 21]. Our findings highlight an important role of calcium entry in cytoskeleton organization and apoptosis and set a stage to the clinical treatment of tumor cell metastasis.

The actin cytoskeleton functions in the generation and maintenance of cell morphology and polarity, in endocytosis and intracellular trafficking, contractility, motility, and cell division. The assembly and disassembly of actin filaments, as well as their organization into functional higher-order networks, are regulated by several extracellular and intracellular signalings [8, 22]. Thapsigargin activates of calcium entry following the depletion of intracellular calcium stores and couples with cytoskeleton organizations. For example, thapsigargin has been reported to induce actin depolymerization and produce a net decrease in F-actin content in human monocytic cells. In turn, prolonged treatment of inhibitors of actin polymerisation abolishes calcium entry by 50% in human platelets [14]. Thus, the coupling of calcium signaling and actin cytoskeletal dynamics need to be further consolidated. Our findings reveal that ectopic opening of calcium influx by thapsigargin may disrupt the organizations of actin cytoskeletons, which may help to understand the relationship of calcium signaling and cytoskeletal dynamics.

It is well established that increased F-actin may promote cell longevity, whereas decreased actin turnover seems to trigger cell death [7]. The actin regulatory protein Cofilin-1 has been shown to have a key role in the apoptotic process. Cofilin-1 is a member of the Cofilin-1/ADF (actin depolymerizing factor) family and regulates actin dynamics by promoting the depolymerization and severing of actin filaments, and regulating the recycling of the resulting monomers. It has been shown that the active (dephosphorylated) form of Cofilin-1 is targeted to mitochondria after initiation of apoptosis. Mitochondrial targeting of Cofilin-1 is sufficient to induce cytochrome c leakage from the mitochondria and strongly induced apoptosis [23]. Moreover, paxillin, a multidomain protein, is one of the key components of for integrin-mediated cytoskeletal reorganization. In tumor cells, paxillin is highly phosphorylated at Tyr118 and recruits other signalling molecules to focal adhesions for tumor metastasis [7, 24]. In the present study, our findings suggested that thapsigargin treatment may dramatically reduce Cofilin-1 phosphorylations and increase its activity (Figures 3(a) and 3(b)), which contribute to the actin depolymerization and initiation of apoptosis. To clarify how thapsigargin inhibits Cofilin-1 phosphorylations, we focus on the mTOR-RhoA pathways. mTOR has been shown to integrate signals from a variety of extracellular inputs, including growth factors, amino acids, glucose, ATP, and oxygen. mTOR-dependent signaling modulates numerous cellular properties, including cell proliferation, cell motility, and protein translation. Inhibition of mTOR kinase activity by rapamycin impairs mTOR-mediated protein synthesis and activities of the small GTPases (e.g., RhoA), leading to inhibition of F-actin organization and cell motility [21, 25]. Moreover, the inhibitory effect of rapamycin on expression of RhoA is also observed in other tumor cell lines, including those derived from cervical cancer (HeLa), prostate cancer (PC-3), Ewing sarcoma (Rh1), and glioblastoma (U-373) [26], suggesting that this is not cell-type dependent. Here, our results suggested that thapsigargin may also down-regualte mTOR kinase activity and inhibit RhoA protein level in A549 human lung adenocarcinoma cells. These findings may contribute to the impairment of actin cytoskeletons by thapsigargin. Thus, our work has set up links of calcium influx, mTOR-RhoA pathways, and cytoskeletal dynamics.

## 5. Conclusion

In summary, the present studies reveal that ectopic calcium influx by thapsigargin inhibits mTOR kinase activity and RhoA expressions, thus leading to the increasing of Cofilin-1 activity and actin depolymerizations. The impairment of actin cytoskeletal dynamics finally triggers cell death in A549 human lung adenocarcinoma cells. Our work suggests that therapies that specifically target calcium-cytoskeleton signaling molecules may prove useful for the treatment of tumor cell metastasis.
